# A simple clinical tool for effective screening of haemophagocytic lymphohistiocytosis in dengue

**DOI:** 10.1007/s15010-025-02542-8

**Published:** 2025-04-22

**Authors:** Jeco Jacob Kuttykandathil, Arfath Ahmed, Gauri Malavalli Girish, Chyavan Trisule Reddy Tummaluru, Vivek Kothandaraman Koushik, Tapendu Patoary, Chakrapani Mahabala

**Affiliations:** 1 Department of General Medicine, Kasturba Medical College Mangalore, Manipal Academy of Higher Education, Manipal, India; 2 Consultant Nephrologist, Prashanth Hospital, Kolathur, Chennai India; 3 Department of Medicine, Ruby General Hospital, Kasba Golpark, Anandapur, Kolkata India

**Keywords:** Severe dengue, Haemophagocytic lymphohistiocytosis, Splenomegaly, Decision tree analysis, Clinical tool

## Abstract

**Purpose:**

Haemophagocytic lymphohistiocytosis (HLH) is a rare, life-threatening disorder. Dengue fever is a common trigger for HLH in the tropics. We aimed to develop a simplified clinical tool to detect HLH in dengue patients.

**Methods:**

A cross-sectional observational study was carried out at Kasturba Medical College Mangalore. Patients between 18 and 60 years of age, with dengue fever for more than five days with suspected HLH symptoms were selected. Hepatosplenomegaly, temperature, haemoglobin levels, total leucocyte count, platelet count, ferritin, triglyceride, and liver function tests were assessed. HLH-2004 criteria were used to confirm the diagnosis. A simple clinical tool was developed via decision tree analysis using clinical and laboratory parameters.

**Results:**

Patients with HLH had marked leucopenia, thrombocytopenia, hyperferritinaemia and elevated aspartate aminotransferase levels, and a greater incidence of hepatosplenomegaly than those without HLH. Decision tree analysis was used to generate a clinical diagnostic tool, which demonstrated an accuracy of 94%, at a confidence interval of 95% (90–98%). The model’s ability to predict HLH was 79%, while its specificity was 96%. It had a positive predictive value of 68% and a negative predictive value of 97%. The kappa value of the predicted model was 0.70, indicating an agreement with the diagnosis using HLH-2004 criteria, with a significant p-value (< 0.001).

**Conclusions:**

Splenomegaly can be used as a screening method to diagnose HLH in patients with dengue. By using an algorithmic approach, combining splenomegaly with leucopenia and thrombocytopenia, this clinical tool accurately detects HLH in patients with dengue.

**Supplementary Information:**

The online version contains supplementary material available at 10.1007/s15010-025-02542-8.

## Introduction

With an estimated annual incidence of 390 million infections worldwide, dengue is the most common arthropod-borne virus that infects humans. A quarter of these infections are symptomatic [1]. Most cases are self-limited, but one in 20 patients with dengue in the United States of America develop severe disease [2]. Over the past decade, the incidence of dengue infection has increased rapidly due to population growth in endemic areas. An estimated sixty per cent of the world’s population could be at risk of dengue infection by 2080 [1]. According to the National Centre for Vector-Borne Disease Control (NCVBDC), there has been steady growth in the annual incidence of dengue in India, with 233,251 cases documented in 2022, rising to 289,000 in 2023 [3].

According to the Intergovernmental Panel on Climate Change (IPCC), climate change facilitates the expansion of vectors and vector-borne diseases, such as dengue, to higher altitudes and latitudes, globalizing the disease. Given the inverse relationship between temperature and elevation, global warming is expected to transform high-altitude ecosystems to resemble those at lower altitudes. In many mountainous regions, glaciers are receding, and cooler-temperature plant species have migrated to higher elevations [4]. Changes in fauna accompany these flora shifts. In Mexico, the first recorded instances of dengue fever at an altitude of 1700 m occurred during an unusually warm summer in 1988 [4]. Changes in the geographic distribution and intensity of malaria and dengue transmission due to future climate change may significantly increase the risk among the immunologically naïve global population and overall disease. Dengue fever is particularly sensitive to climatic variations, with optimal transmission at 27 to 35 degrees Celsius. Meteorological conditions affect mosquito longevity, parity, fecundity, gonotrophic cycle, and incubation periods, influencing disease transmission. Temperature and humidity impact dengue by affecting larval development, adult feeding behaviour, and mosquito survival, while rainfall creates breeding habitats. However, excessive rainfall can disrupt breeding sites, leading to varied associations between rainfall and dengue incidence [5–9].

The strain of the virus, the host immune response, and genetic factors play vital roles in disease pathogenesis and severity. Severe dengue is noted to have increased plasma leakage and abnormal haemostasis. An uncontrolled inflammatory response with high ferritin levels is a rare complication of dengue. Recent studies have shown that ferritin is a valuable marker for inflammation in dengue [10].

Haemophagocytic lymphohistiocytosis (HLH) is a life-threatening monocyte/ macrophage-related histiocytic disorder. There are familial and sporadic variants of this disease. It is characterised by hyperinflammation, which can cause cytopenia(s), splenomegaly, hepatitis, and central nervous system dysfunction. Farquhar and Claireux first described HLH in 1952 as a familial disease [11]. Identifying HLH is challenging because of its overlapping features with other inflammatory disorders, hence the need for a simple diagnostic clinical tool. Thus, the exact incidence and prevalence of HLH are challenging to determine but are estimated to occur in 1 in 2000 adult admissions in tertiary care centres [12].

Sporadic HLH, previously referred to as secondary HLH, is triggered by infection, malignancy, rheumatologic disorders, immune-activating therapies, and immune suppression. Infections such as Ebstein-Barr virus (EBV), influenza virus, cytomegalovirus (CMV), and in endemic areas, dengue, malaria, toxoplasmosis, and leishmaniasis can trigger HLH [12]. 

According to recent studies, 3.12% of patients with dengue fever develop HLH, which increases to 10 − 22.1% in severe dengue [13]. In dengue-associated HLH, dengue infection triggers macrophage activation, leading to cytokine storm and immune-mediated tissue destruction [13, 14]. Mortality in dengue-associated HLH patients is reported to be as high as 20.2% [13].

Fever, cytopenia(s), hepatomegaly, hepatitis and hyperferritinaemia occur both in severe dengue and HLH patients. In addition to these features, patients with HLH have prolonged fever (lasting more than seven days), splenomegaly, elevated lactate dehydrogenase (LDH) and triglyceride levels, hypofibrinogenaemia and haemophagocytosis [15]. A high degree of clinical suspicion is required to diagnose HLH in the setting of severe dengue.

The H-score is used to diagnose secondary HLH in dengue patients and includes parameters such as ferritin, triglycerides, liver enzymes, and fibrinogen [16]. A complete battery of these tests, including ferritin and CD-25, is expensive and inaccessible in resource-limited settings. To effectively screen patients for HLH, it is prudent to utilise a clinical tool, which leads to a high pretest probability for definitive tests and low false negative results.

A recent study has shown that the soluble IL-2 receptor (sIL-2R), which corresponds to T-cell activation, is a better predictor of HLH than serum ferritin, which is regularly used as one of the key markers to diagnose HLH [17].

This study aims to develop a simplified clinical tool that formulates specific criteria for screening dengue patients for HLH. This tool aims to ensure that hospital resources are utilised effectively and reduce the financial burden on the patients, who could otherwise not afford a comprehensive set of diagnostic tests in resource-limited settings.

## Materials and methods

A cross-sectional observational study was conducted at the teaching hospitals affiliated with Kasturba Medical College Mangalore, among patients diagnosed with dengue, with fever lasting for more than five days, and with clinical suspicion of HLH. Dengue was diagnosed with a rapid antigen test and enzyme-linked immunosorbent assay (ELISA). In this study, we included patients experiencing their first episode of dengue fever. The study protocol was approved by the Institutional Ethics Committee (IEC) of Kasturba Medical College Mangalore (IEC KMC MLR 09–16/228), complying with the guidelines of the Declaration of Helsinki.

We included patients aged 18–60 years after informed written consent and excluded those with liver disease, malignancy, thalassemia, pregnancy, rheumatological disorders, nephrotic syndrome, and those who had received hormonal therapy. Other causes of HLH were excluded.

Data was collected from 173 patients who met the inclusion criteria and was anonymised. The temperature, haemoglobin, total leucocyte count, platelet count, ferritin levels, triglyceride levels, alanine transaminase (ALT) levels, and aspartate transaminase (AST) levels of each patient were collected. Patients were examined for hepatosplenomegaly. Serum fibrinogen, NK cell activity, soluble CD-25, and bone marrow biopsy were not performed.

According to the HLH-2004 criteria, the diagnosis of HLH can be confirmed if one of the following conditions is met [18]:

1. A molecular diagnosis consistent with HLH Or.
2. The diagnostic criteria for HLH is satisfied, specifically five out of eight criteria, listed below:
  - Initial diagnostic criteria (to be assessed in all patients with HLH).
    i. Fever.
    ii. Splenomegaly.
    iii. Cytopenia affecting two of the three lines in the peripheral blood:
      Haemoglobin < 90 g/L (an infant less than four weeks: haemoglobin < 100 g/L).
      Platelets < 100 × 10^9^/L
      Neutrophils < 1.0 × 10^9^/L
    iv. Hypertriglyceridemia and/or hypofibrinogenaemia:
      Fasting triglycerides ≥ 3.0 mmol/L (i.e., 265 mg/dL).
      Fibrinogen ≤ 1.5 g/L.
    v. Haemophagocytosis in bone marrow, spleen, or lymph nodes.
    vi. Absence of evidence for malignancy.
    b. The following three parameters were added to the criteria in the year 2004.
    i. Low or absent natural killer (NK) cell activity (according to local laboratory reference).
    ii. Ferritin ≥ 500 mg/L.
    iii. Soluble CD25 (i.e., soluble IL-2 receptor) ≥ 2400 U/mL.

Decision tree analysis of the data was performed via classification and regression tree analysis, the quick, unbiased, efficient statistical tree (QUEST) method. The HLH-2004 criteria were used as a dependent variable, using IBM SPSS Statistics for Windows, version 25.0 (IBM Corp., Armonk, NY, USA) to create a simplified clinical tool for diagnosing HLH.

Following the formulation of the clinical tool, it was assessed for sensitivity, specificity, positive and negative predictive values, and accuracy.

Sensitivity (True Positive Rate): Sensitivity measures the proportion of actual positives (HLH positive) that are correctly identified by the test, i.e., Sensitivity = True Positives (TP) /Total HLH Positive.

Specificity (True Negative Rate): Specificity measures the proportion of actual negatives (HLH negative) that are correctly identified by the test, i.e., Specificity = True Negatives (TN) / Total HLH Negative.

Positive Predictive Value (PPV): PPV measures the proportion of positive test results that are true positives, i.e., Positive Predictive Value = True Positives (TP) / Total Positive Test Results.

Negative Predictive Value (NPV): NPV measures the proportion of negative test results that are true negatives, i.e., Negative Predictive Value = True Negatives (TN) / Total Negative Test Results.

Accuracy: Accuracy measures the proportion of true results (both true positives and true negatives) among the total number of cases examined.

Accuracy = [True Positives (TP) + True Negatives (TN)] / Total Number of Cases; or,

Accuracy = [True Positives (TP) + True Negatives (TN)] / [TP + TN + False Positives (FP) + False Negatives (FN)]

## Results

One hundred and seventy-three patients diagnosed with dengue fever lasting more than five days were analysed. Among the 173 patients evaluated, 19 individuals (11%) met the HLH-2004 criteria and were classified as HLH-positive.

The two groups showed no significant difference in age, maximum temperature, least haemoglobin level, or serum triglyceride level, as shown in Table 1.

**Table 1:** A comparative analysis of clinical and laboratory characteristics of HLH in dengue patients

Parameters	HLH positive (*n* = 19) Median, (IQR), [Range]	HLH negative (*n* = 154) Median, (IQR), [Range]	*p*-value
Age (years)	35.0 (24–47) [16–55]	38.0 (25.75-49.0) [15–85]	0.681
Maximum temperature (°C)	39.8 (39.7–39.9) [39.5–40]	39.8 (39.6–39.9) [38.5–41.0]	0.243
Least haemoglobin (g/L)	146 (121–160) [84–176]	136 (120.7–146.0) [85–182]	0.095
Least total leucocyte count (x10^9^ cells/L)	2.50 (2.0-3.40) [0.80-5.0]	3.80 (3.07-5.0) [0.70–10.4]	< 0.001
Least platelet count (x10^9^ cells/L)	15 (12–20) [4–55]	24 (15–67) [6-234]	0.002
Ferritin (µg/L)	13,295 (9536–17374) [7360–65177]	2552 (835–5608) [19.60- 42650]	< 0.001
Triglycerides (mmol/L)	2.39 (1.55–3.38) [0.41–4.52]	2.05 (1.50–2.76) [0.83–3.39]	0.352
AST (U/L)	324 (174–497) [76-1470]	117 (73–209) [19-3975]	< 0.001
Neutrophil count (x10^9^ cells/L)	1.62 (1.10–2.28) [0.10–3.90]	2.21 (1.55–3.08) [0.29–8.88]	0.011
Hepatomegaly (*n*)	17	78	0.001
Splenomegaly (*n*)	19	76	< 0.001

Note: Age is presented in years. Maximum temperature is in degrees Celsius (°C). Least haemoglobin is expressed in grams per litre (g/L). Least total leucocyte count and least platelet count are expressed as cells x10⁹/Litre. Ferritin levels are in micrograms per litre (µg/L). Triglycerides are in millimoles per litre (mmol/L). Aspartate aminotransferase (AST) is in units per litre (U/L). Hepatomegaly and splenomegaly are reported as the number of cases (n)

Patients with dengue fever fulfilling the HLH-2004 criteria had lower platelet and total leucocyte counts, higher ferritin and AST levels, and a greater incidence of hepatosplenomegaly than HLH-negative patients.

**Fig. 1:**
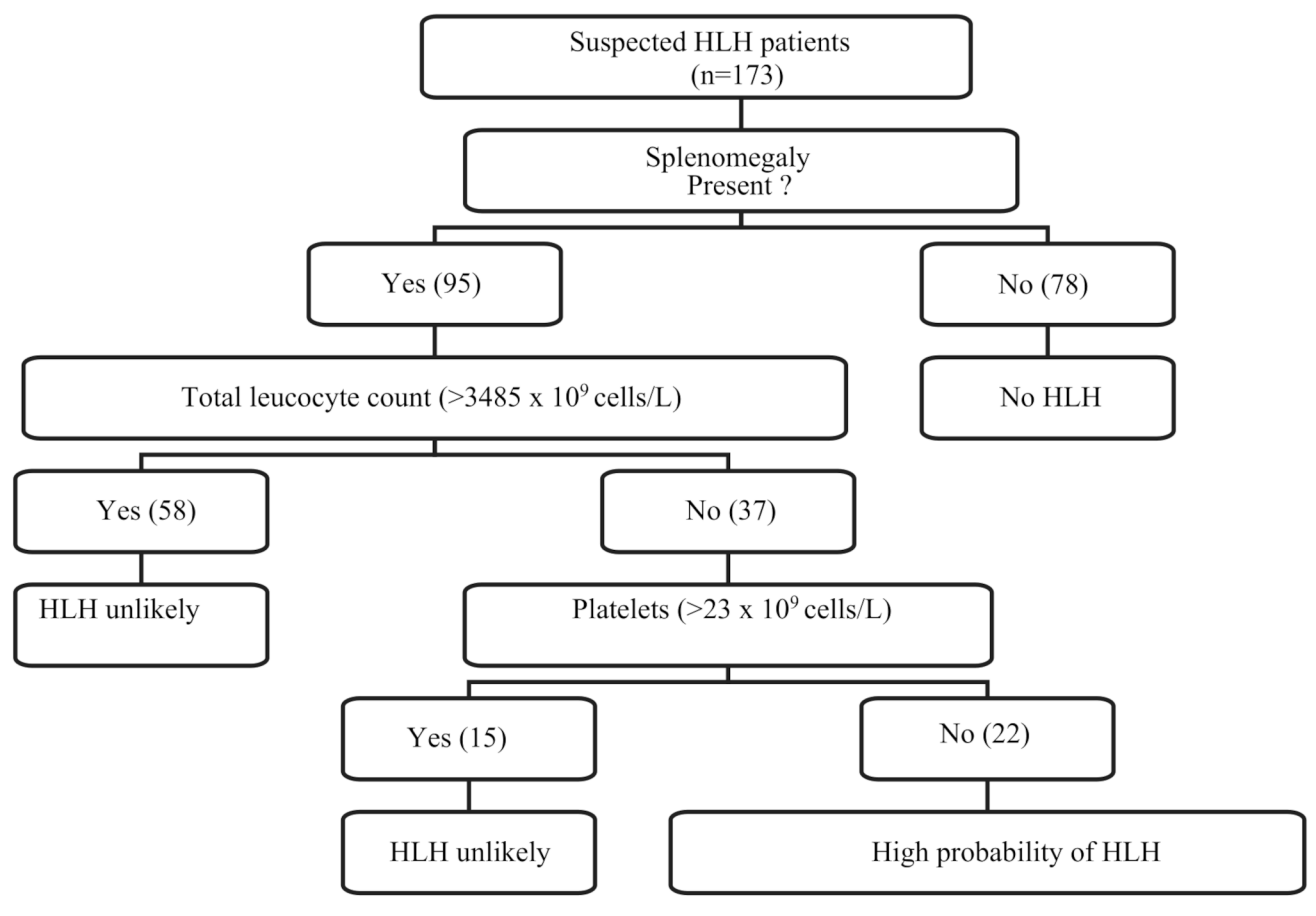
Utilizing Splenomegaly, Leucocyte, and Platelet Counts to Assess HLH Risk in Dengue Patients: A Stepwise Diagnostic Approach

The new clinical tool obtained via the QUEST method was developed based on decision tree analysis, which consists of three parameters: splenomegaly, low total leucocyte count, and low platelet count (Fig. 1). Table 2 shows the distributions of these three parameters.

**Table 2:** Comparative distribution of the three selected parameters among the HLH-2004 positive and HLH-2004 negative cases

	HLH Negative (*n* = 154)	HLH Positive (*n* = 19)	Total (*n* = 173)
**Splenomegaly Positive** (n)	76 (80%)	19 (20%)	95
**Splenomegaly Negative** (n)	78 (100%)	0 (0%)	78
**Median total leucocyte count ( < = 3.48579)** (cells x10^9^/L)	22 (59.5%)	15 (40.5%)	37
**Median total leucocyte count (> 3.485)** (cells x10^9^/L)	54 (93.1%)	4 (6.9%)	58
**Median platelet count ( < = 23.75950)** (cells x10^9^/L)	7 (31.8%)	15 (68.2%)	22
**Median platelet count (> 23.75950)** (cells x10^9^/L)	15 (100%)	0 (0%)	15

Note: Splenomegaly positive and splenomegaly negative are reported as the number of cases (*n*). Total leucocyte count is expressed as cells x10⁹/litre, with values ≤ 3.48579 and > 3.485 separated. Platelet count is reported as cells x10⁹/litre, with values ≤ 23.75950 and > 23.75950 separated

### Cross-validation

**Table 3:** Contingency table for calculating diagnostic accuracy metrics of the proposed clinical tool

Simplified clinical tool		HLH Positive (*n* = 19)	HLH Negative (*n* = 154)
	**Positive as per the tool**	True positives 15	False positives 7
	**Negative as per the tool**	False Negative 4	True Negative 147

Note: True Positive (TP): Number of cases where the tool correctly identified HLH. False Positive (FP): Number of cases where the tool incorrectly identified HLH. True Negative (TN): Number of cases where the tool correctly identified HLH. False Negative (FN): Number of cases where the tool incorrectly identified HLH

The sensitivity of this model in predicting HLH was 79%, and the specificity was 96%, with a positive predictive value of 68% and a negative predictive value of 97% (Table 3).

The kappa value of the predicted model was 0.7, which strongly agrees with the actual HLH-2004 criteria, and a p-value < 0.001 indicates that the study is statistically significant.

In our study, all participants recovered, with the exception of one unfortunate patient who suffered from HLH.

## Discussion

Hemophagocytic lymphohistiocytosis is a rare but serious complication of dengue infection that primarily affects infants but can also affect children and adults [19]. Symptoms include fever, rash, hepatomegaly, splenomegaly, jaundice, and lymphadenopathy. It can also lead to multiorgan failure. Fever in HLH is due to high interleukin levels, whereas infiltration of the spleen by lymphocytes and macrophages causes splenomegaly. Haemophagocytosis by macrophages in the bone marrow leads to cytopenia(s). Additionally, hypertriglyceridaemia is caused by the decreased activity of lipoprotein lipase [20].

Our study prioritises the accurate detection of HLH occurring in dengue. According to the Histiocyte Society, the criteria used to diagnose HLH include fever, splenomegaly, cytopenia, hypertriglyceridaemia, hypofibrinogenaemia, and haemophagocytosis [21]. However, three more criteria were added in 2004, i.e. hyperferritinaemia, low or absent NK cell activity, and high-soluble interleukin-2 receptor levels [18, 22]. Five out of the above eight criteria must be present to make a diagnosis of HLH.

Among the 173 patients with dengue, 19 (11%) were found to have HLH according to the HLH-2004 criteria, and 89% of the total patients tested were negative for HLH. We observed that splenomegaly, a chief symptom of HLH, could be used as a primary factor for initial screening and testing. Among the 173 dengue patients screened, 78% did not have splenomegaly. These patients could have been exempt from an initial battery of tests, reducing their financial burden. Ninety-five patients were positive for splenomegaly, and all 19 patients with HLH had splenomegaly. This finding supports our hypothesis that splenomegaly should be one of the first signs screened for in dengue patients with suspected HLH and can also be used as a single effective marker to diagnose the disease. Another study in Puerto Rico between 2008 and 2013 revealed that out of 22 confirmed cases of dengue-associated HLH, 17 (77.3%) had splenomegaly, further supporting our theory for early detection of HLH using splenomegaly. An early diagnosis of splenomegaly would raise suspicions of HLH in clinicians, facilitating earlier treatment and reducing progression to multiorgan failure, as seen in various case studies [23].

In our study, low total counts were also associated with HLH. Patients with a total leucocyte count > 3.485 × 10^9^ cells/L had a lower probability of HLH. Among the 58 patients with total leucocyte counts above 3.485 × 10^9^ cells/L, only 6% had HLH, whereas 40.5% of patients with low total leucocyte counts (<3.485 × 10^9^ cells/L) had HLH. This correlation was also observed in a case report from Eastern India in 2017, where patients clinically diagnosed with dengue-associated HLH had persistently low total leucocyte counts [24].

The 37 patients with low total leucocyte counts were further studied, and their platelet counts were tested for comparison. A low platelet count of < = 23.75950 × 10^9^ cells/L was found to be significant in patients with HLH. We found that 22 patients had a lower platelet count, and 15(68.2%) had HLH. Only seven (31.8%) patients were negative for HLH. All patients with a platelet count of> 23.75950 × 10^9^ cells/L tested negative for HLH. Therefore, splenomegaly, low total leucocyte counts, and low platelet counts can be used for primary screening in dengue patients suspected of having HLH.

According to the study model, the dengue patients who had splenomegaly were tested for platelet and total leucocyte counts. Further testing would be advised based on their results, which would include ferritin, triglycerides, and AST in a confirmatory group of tests, thus suggesting the use of a simplified clinical tool for testing for HLH in dengue patients.

This model predicted that splenomegaly could be an effective marker for diagnosing HLH in dengue patients. Although fever and high ferritin levels were more sensitive indicators of the disease, we observed that all 19 patients with HLH presented with splenomegaly. Low platelet and total leucocyte counts are also valuable predictors of HLH. However, we recognise that these factors are present in a patient suffering from severe dengue, thus further stressing the presence of splenomegaly as an exclusive and efficient marker for diagnosing the disease. This would be cost-effective in a resource-limited setting. We further suggest that splenomegaly could also be used as a single marker to start first-line treatment for dengue-associated HLH in these patients, but a larger sample size is needed to support these observations. Testing other parameters, such as ferritin and CD25, is warranted only on a confirmatory basis.

Bone marrow studies are not recommended for diagnosing dengue-associated HLH, as prior studies have indicated that bone marrow biopsies performed early in the disease course may be normal [25].

In managing dengue-associated HLH, it is crucial to monitor patients for indications of cytokine storm and multi-organ dysfunction, given the significant mortality risk associated with this condition [26]. Early intervention requires treatment based on strong clinical suspicion rather than complex diagnostic procedures. Soluble interleukin-2 receptor (sIL-2R) levels can be used as a biomarker to effectively identify patients at risk of progressing to HLH, facilitating targeted and timely treatment decisions. This approach enables healthcare providers to prioritise individuals with elevated sIL-2R, enhancing patient outcomes and optimising the management of this condition [17].

In the absence of randomised trials for the treatment of dengue-associated HLH, the selection of appropriate therapeutic interventions remains uncertain and challenging. Existing literature emphasises supportive care with or without short courses (3–4 days) of high-dose corticosteroids. Although certain patients have demonstrated positive outcomes in the absence of corticosteroid therapy, the significant mortality associated with HLH and the relatively low risk linked to brief corticosteroid regimens justifies their use. Intravenous administration of specific corticosteroids, such as methylprednisolone or dexamethasone, is recommended. Dexamethasone is particularly favoured for cases involving the central nervous system due to its enhanced ability to penetrate the blood-brain barrier.

Furthermore, the use of a combined therapeutic approach involving corticosteroids and immunoglobulin has been suggested. It is generally accepted that the treatment of dengue-associated HLH does not include chemotherapy or hematopoietic stem cell transplantation. It is crucial to promptly address bacterial coinfections in these patients, which occur in 33% of cases, to ensure comprehensive patient management [25].

## Limitations

Our study had a small sample size and was a single-centric study with only internal validation in the form of cross-validation in the statistical analysis. We require a larger multicentric study with external validation to confirm these observations.

## Conclusion

Our study revealed that splenomegaly could be an effective marker for diagnosing HLH in dengue patients. It forms the basis of our simplified clinical tool, which includes two other factors – low total leucocyte counts, and low platelet counts. This tool is designed for resource-limited settings and countries with a high incidence of dengue infections. This approach facilitates a quicker and more accurate diagnosis with a low chance of false negatives, which is essential for further treatment.

## Electronic supplementary material

Below is the link to the electronic supplementary material.

Supplementary Material 1

## Data Availability

Data is provided within the manuscript or supplementary information files.

## Abbrevations

HLH: Haemophagocytic lymphohistiocytosis
NCVBDC: National Centre for Vector-Borne Disease Control
EBV: Ebstein-Barr virus
CMV: Cytomegalovirus
LDH: Lactate dehydrogenase
CD-25: Cluster of Differentiation 25
sIL-2R: Soluble Interleukin-2 receptor
ELISA: Enzyme-linked immunosorbent assay
IEC: Institutional Ethics Committee
ALT: Alanine transaminase
AST: Aspartate transaminase
NK: Natural killer
QUEST: Quick, unbiased, efficient statistical tree
